# Diagnostic performance of PI-RADS version 2.1 compared to version 2.0 for detection of peripheral and transition zone prostate cancer

**DOI:** 10.1038/s41598-020-72544-z

**Published:** 2020-09-29

**Authors:** Madhuri Monique Rudolph, Alexander Daniel Jacques Baur, Hannes Cash, Matthias Haas, Samy Mahjoub, Alexander Hartenstein, Charlie Alexander Hamm, Nick Lasse Beetz, Frank Konietschke, Bernd Hamm, Patrick Asbach, Tobias Penzkofer

**Affiliations:** 1 Department of Radiology, Charité – Universitätsmedizin Berlin, Freie Universität Berlin, Humboldt-Universität zu Berlin and Berlin Institute of Health, Augustenburger Platz 1, 13353 Berlin, Germany; 2 Department of Urology, Charité – Universitätsmedizin Berlin, Freie Universität Berlin, Humboldt-Universität zu Berlin and Berlin Institute of Health, Charitéplatz 1, 13353 Berlin, Germany; 3 Department of Urology, Cologne University Hospital, 50937 Cologne, Germany; 4 Institute of Biometry and Clinical Epidemiology, Charité – Universitätsmedizin Berlin, Freie Universität Berlin, Humboldt-Universität zu Berlin and Berlin Institute of Health, 10117 Berlin, Germany; 5 Berlin Institute of Health (BIH), Anna-Louisa-Karsch-Str. 2, 10178 Berlin, Germany

**Keywords:** Prostate cancer, Prostate, Prostate cancer

## Abstract

The purpose of this study is to compare diagnostic performance of Prostate Imaging Reporting and Data System (PI-RADS) version (v) 2.1 and 2.0 for detection of Gleason Score (GS) ≥ 7 prostate cancer on MRI. Three experienced radiologists provided PI-RADS v2.0 scores and at least 12 months later v2.1 scores on lesions in 333 prostate MRI examinations acquired between 2012 and 2015. Diagnostic performance was assessed retrospectively by using MRI/transrectal ultrasound fusion biopsy and 10-core systematic biopsy as the reference. From a total of 359 lesions, GS ≥ 7 tumor was present in 135 lesions (37.60%). Area under the ROC curve (AUC) revealed slightly lower values for peripheral zone (PZ) and transition zone (TZ) scoring in v2.1, but these differences did not reach statistical significance. A significant number of score 2 lesions in the TZ were downgraded to score 1 in v2.1 showing 0% GS ≥ 7 tumor (0/11). The newly introduced diffusion-weighted imaging (DWI) upgrading rule in v2.1 was applied in 6 lesions from a total of 143 TZ lesions (4.2%). In summary, PI-RADS v2.1 showed no statistically significant differences in overall diagnostic performance of TZ and PZ scoring compared to v2.0. Downgraded BPH nodules showed favorable cancer frequencies. The new DWI upgrading rule for TZ lesions was applied in only few cases.

## Introduction

The prostate imaging and reporting data system (PI-RADS) was created to standardize prostate MRI reporting with the goals of improving prostate cancer detection in MRI and achieving a more structured and comparable reporting quality^1,2^. The standard is based on the consensus of a group of experts incorporating scientific evidence and their experience in prostate MRI. In 2012 the first version of PI-RADS was published^1^, and in late 2014 PI-RADS v2.0 was released^2^. PI-RADS has been rapidly accepted among the radiological and urological community and is being incorporated into clinical care pathways^3^, as many studies have shown good diagnostic accuracy of the reporting system^4–8^.

In March 2019, modifications to PI-RADS version 2, termed PI-RADS v2.1, were published with carefully selected improvements and the assertion that further research was required to corroborate the incremental value^9^. PI-RADS v2.1 includes modifications to technical specifications as well as revisions in the scoring of prostate lesions. The scoring of transition zone (TZ) lesions has changed with regards to scores 1–3: typical benign prostatic hyperplasia (BPH) nodules are now scored as T2 weighted (T2W) score 1 as opposed to previously score 2. Atypical nodules, that are mostly encapsulated or homogenous without encapsulation, are now scored as T2W score 2 as opposed to previously score 3. Furthermore, a TZ lesion with a T2W score of 2 can be upgraded to an overall score of 3 when a DWI score of 4 or 5 is assigned. Another important change concerns DWI scores 2 and 3 for all zones: a linear or wedge-shaped ADC-hypointense or DWI-hyperintense lesion should be scored as score 2 now, whereas formerly indistinct ADC-hypointense lesions were scored into this category. The criteria for DWI score of 3 have been slightly modified: in PI-RADS v2.1 focal ADC-hypointense and/or focal DWI hyperintense lesions are scored a 3. Signal intensities can be marked in one of the sequences but should not be marked in both sequences. The definition of “marked” has been clarified as “a more pronounced signal change than any other focus in the same zone”^9^. With these updates in the scoring system, changes in the diagnostic accuracy of the scoring system are possible.

The purpose of this study was to directly compare diagnostic performance of PI-RADS v2.0 and v2.1 for detection of GS ≥ 7 tumor using targeted MRI/transrectal ultrasound fusion biopsy (TB) and systematic 10-core biopsy as a reference standard.

## Methods

### Patient cohort

**Figure 1:**
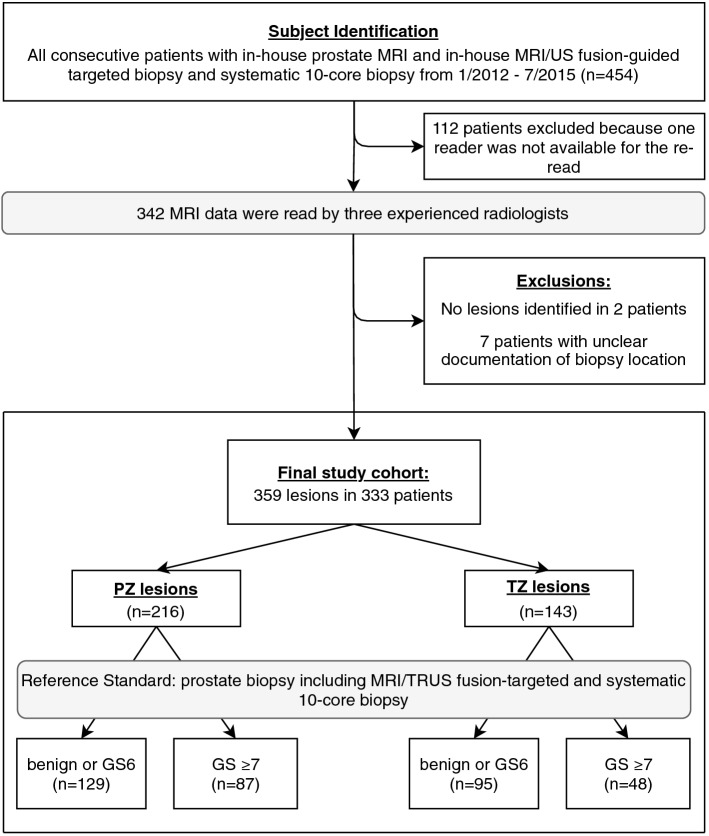
Study workflow with patient population. *PZ* peripheral zone, *TZ* transition zone, *TRUS* transrectal ultrasound, *GS* Gleason Score.

This retrospective, single-center study was approved by the institutional review board (Ethikkommission der Charité – Universitätsmedizin Berlin) and patient consent was waived by the latter. All methods were carried out in accordance with relevant guidelines and regulations. All patients who received prostate MRI and subsequent MRI/TRUS fusion prostate biopsy in combination with 10-core systematic biopsy at our institution between January 2012 and July 2015 were considered eligible for this study (n = 454). Exclusion criteria were incomplete or non-standard MRI. Subgroups of the same collective have been included in earlier studies with endpoints independent from this study^10–16^. Patients were assigned randomly to initially four readers, 112 to 114 lesions by each radiologist. One reader was not available for the re-read after 12 months. The remaining 342 patients were re-read, 9 patients were then excluded because of unclear documentation of the targeted lesion’s location in TB (n = 7) or because no lesion was identified by the reader (n = 2), leaving a final study cohort of 333 patients. Figure 1 contains a STARD 2015-compliant patient flow diagram.

### MR imaging

MRI was acquired according to relevant ESUR guidelines. All imaging was performed on one of two identical 3T MRI scanners (Skyra, Siemens Healthineers, Erlangen, Germany). All patients received at least biparametric MRI including T2W and DW images. In 186 patients (54.4%) DCE was performed additionally. Typical parameters were: Axial and coronal T2W imaging (3.0 × 0.47 × 0.47 mm, 18 cm FoV), axial diffusion-weighted imaging (3.0 × 1.4 × 1.4 mm, 17 cm FoV, with b-values of 0, 50, 500, 800/1000 or calculated b = 1400 s/mm^2^). Axial T1-weighted imaging (3.0 × 0.6 × 0.6 mm, 32 cm FoV) of the whole pelvis and axial dynamic contrast enhanced imaging (3.0 × 1.4 × 1.4 mm, 18.6 cm FoV, at a temporal resolution of 5 s, 3 ml/s injection flow, Gd-DO3A-butrol, Gadovist, Bayer Healthcare, Leverkusen, Germany).

### Imaging review

342 MRI datasets were divided into equally sized subgroups and assigned to one of three board certified radiologists with extensive experience in prostate MRI (P.A., A.B, M.H., all with > 5 years of experience in prostate MRI). Reviewing was performed in a blinded and randomized setting by using a standardized, in-house built reviewing software. Readers were blinded to histopathological results and all other patient-related data. In the first round, readers were instructed to mark dominant prostate lesions, to assign DWI-, T2W- and DCE-scores according to PI-RADS v2.0 guidelines, and to tag the localization of the lesion according to the segmentation model of PI-RADS v2.0. For MRI datasets without DCE images lesions were scored according to the rules for assessment without adequate DCE as specified in the PI-RADS v2.0 guidelines. At least 12 months later, the previously marked MRI datasets were presented to the same readers. Readers assessed the same patients as in the previous session. They were blinded to their previous assessment and were instructed to assign PI-RADS v2.1 scores to every lesion they had marked before as well as identify the matching v2.1 segments. This led to a 1:1 comparison of PI-RADS v2.0 and v2.1 for the same reader on a per lesion basis. The overall score per lesion was assigned in accordance with the respective algorithm for PI-RADS v2.0 or v2.1, following the dominant sequence and upgrading rules. Furthermore, lesions were attributed to either the peripheral zone (PZ) or the transition zone (TZ). Lesions that extended through PZ as well as TZ and lesions that were located in the anterior stroma (AS) or central zone (CZ) were assigned to either the PZ or the TZ group depending on the most likely zone of origin.

### Reference standard

All patients had undergone targeted MRI/TRUS fusion biopsy (TB) in combination with a 10-core systematic biopsy in the same session. Histopathological findings of cancerous lesions were classified according to the Gleason grading system. A GS of 3 + 4 or higher on TB or in a matching segment on systematic biopsy was considered positive for clinically significant prostate cancer (csPCa). If no cancerous tissue was found upon TB or systematic biopsy in the segment of the suspicious MR lesion, the respective lesion was considered negative for PCa. Histopathological findings that indicated non-cancerous changes were: no tumor cells, acute prostatitis, chronic prostatitis, granulomatous prostatitis, prostatic intraepithelial neoplasia or benign prostatic hyperplasia, each without the additional mentioning of a neoplastic disease.

### Statistical analysis

Data was analyzed on a per lesion basis and was modeled in terms of a factorial diagnostic trial involving the factors “scoring system” (PI-RADS v2.0 versus PI-RADS v2.1) and “reader”. The area under the ROC curve (AUC) of each reader and scoring system combination assessed the diagnostic accuracy. Since data are measured on an ordinal scale, we applied the nonparametric ANOVA-type statistic^17^ to test differences between the AUC of the two scoring systems and the three readers as well as interactions between readers and scoring systems^18^. Proportions of cancerous lesions per score were compared using the two-proportions Z-Test. Sensitivity, specificity, positive predictive value (PPV) and negative predictive value were calculated after dichotomizing the PI-RADS assessment categories using a predefined cut-off value: a PI-RADS category of ≥ 3 was defined as positive. Sensitivity and specificity of the two versions were compared using the McNemar’s test. PPV and NPV were compared using the test by Lange and Brunner^19^.

Results were declared to be significant if *p* < 5%. Statistical analysis was performed by F.K. and T.P. using R version 1.1.419 (www.r-project.org) and SAS version 9.4.

## Results

### Patient and lesion characteristics

The final study cohort consisted of 333 patients with a mean age of 66.8 years and a mean PSA level of 12.8 ng/ml. Patient characteristics are summarized in Table 1. Readers marked 359 lesions in the 333 MRI datasets. GS ≥ 6 tumor was detected in 193 (53.8%) lesions, including GS ≥ 7 tumor in 135 (37.6%) lesions. In the PZ, 216 (60.2%) lesions were marked; prevalence of GS ≥ 7 tumor in PZ lesions was 87 (40.3%). In the TZ, 143 lesions (39.8%) were marked; prevalence of GS ≥ 7 tumor in TZ lesions was 48 (33.6%).

**Table 1:** Patient characteristics.

Parameter	Value ± standard deviance [min–max]
Number of patients	333
Age (years)	66.8 ± 7.4 [47–85]
PSA (ng/ml)	12.8 ± 11.7 [1.47–112]
Prostate volume (ml)	62.8 ± 32.1 [6.10–203.66]
**Biopsy result (highest grade)**	
No cancer nor inflammation	56 (16.8%)
Inflammation (chronic or acute)	51 (15.3%)
PIN	10 (3.0%)
3 + 3	64 (19.2%)
3 + 4	44 (13.2%)
4 + 3	31 (9.3%)
4 + 4	57 (17.1%)
4 + 5	13 (3.9%)
5 + 4	4 (1.2%)
5 + 5	3 (0.9%)

Values are given as mean ± standard deviation [range] for continuous variables and absolute frequency (relative frequency) for biopsy results.

*PSA* prostate-specific antigen, *PIN* prostatic intraepithelial neoplasia.

### Head to head comparison and statistical interactions

**Figure 2:**
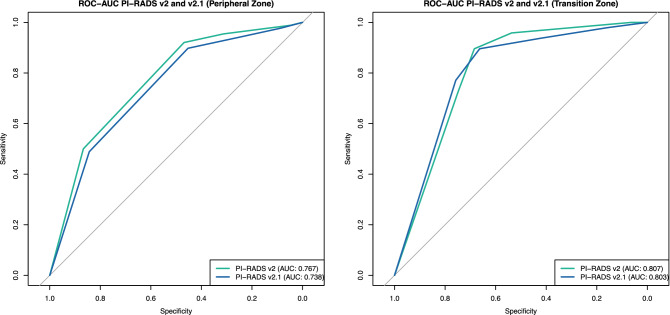
ROC for Gleason Score ≥ 7 tumor detection in PI-RADS v2.0 and v2.1 regarding overall PI-RADS scores per lesion. Differences in area under the ROC curves (AUC) were not significant. *v* version.

Diagnostic performance of the scoring systems was assessed by ROC curves and AUC. ROC-Curves for the overall score of PZ and TZ lesions are given in Fig. 2. ROC-Curves and AUC values separated by readers are given in the supplemental material (supplemental Table 1 and supplemental Figure 1). Evaluation with and without DCE did not yield significant differences in AUC for both PI-RADS v2.0 and v2.1 (supplemental Table 2 and supplemental Figure 2).

For the PZ, ROC analysis revealed a slightly higher AUC for v2.0 (AUC 0.767 in v2.0 vs. AUC 0.738 in v2.1; *p* > 0.05), but the difference did not reach statistical significance. In TZ lesions, the AUC was also slightly higher for PI-RADS v2.0 (AUC 0.807 in v2.0 vs. AUC 0.803 in v2.1; *p* > 0.05), again the difference did not reach statistical significance. Furthermore, no significant difference between performances of the three readers and no interactions between readers and scoring systems could be detected using the nonparametric ANOVA-type statistic (*p* > 0.05)^17^.

**Figure 3:**
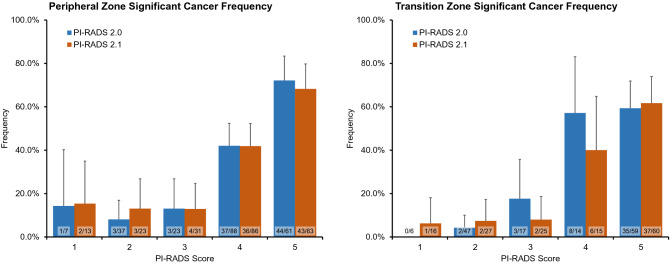
Frequency of significant cancer defined as Gleason Score (GS) ≥ 7 tumor separated by zone (PZ left, TZ right), compared by version (blue: PI-RADS v2.0, orange: PI-RADS v2.1) and score (x-axis). Differences in GS ≥ 7 tumor frequencies were not significant.

Frequency of assigned scores are compared in Table 2. In PI-RADS v2.1, significantly more TZ lesions were assigned an overall score of 1 (4.2% in v2.0 vs. 11.2% in v2.1, *p* = 0.046) while significantly less TZ lesions were assigned an overall score of 2 (32.9% in v2.0 vs. 18.9% in v2.1, *p* = 0.010). In both zones, more lesions were assigned an overall score of 3 in v2.1, but the difference was not statistically significant. The frequency of GS ≥ 7 tumor per score did not differ significantly between the two versions (Table 2, Fig. 3). Frequency of GS ≥ 7 tumor for scores 4 and 5 were high in both versions, ranging from 40 to 57.1% (PI-RADS 4) and 59.3% to 72.1% (PI-RADS 5), respectively. For scores 1 to 3 frequency of GS ≥ 7 tumor ranged from 0 to 18.8% in both versions.

**Table 2:** Frequencies of assigned scores and frequencies of Gleason Score ≥ 7 tumor per overall PI-RADS score in peripheral and transition zone lesions.

Zone	Overall Score	Frequency of assigned score			Frequency of GS ≥ 7 tumor		
		PI-RADS v2.0	PI-RADS v2.1	*p* value	PI-RADS v2.0	PI-RADS v2.1	*p* value
PZ	1	3.2% (7/216)	6.0% (13/216)	0.25	14.3% (1/7)	15.4% (2/13)	1.00
	2	17.1% (37/216)	10.6% (23/216)	0.071	8.1% (3/37)	13.0% (3/23)	0.86
	3	10.6% (23/216)	14.4% (31/216)	0.31	13.0% (3/23)	10.0% (3/30)	1.00
	4	40.7% (88/216)	39.8% (86/216)	0.92	42.4% (36/85)	42.9% (36/84)	1.00
	5	28.2% (61/216)	29.2% (63/216)	0.92	72.1% (44/61)	68.3% (43/63)	0.78
TZ	1	4.2% (6/143)	11.2% (16/143)	0.046*	0.0% (0/6)	6.3% (1/16)	1.00
	2	32.9% (47/143)	18.9% (27/143)	0.010*	4.3% (2/47)	7.4% (2/27)	0.97
	3	11.9% (17/143)	17.5% (25/143)	0.24	18.8% (3/16)	8.3% (2/24)	0.64
	4	9.8% (14/143)	10.5% (15/143)	1.00	57.1% (8/14)	40.0% (6/15)	0.58
	5	41.3% (59/143)	42.0% (60/143)	1.00	59.3% (35/59)	61.7% (37/60)	0.94

Significantly more TZ lesions were assigned to category 1 in v2.1 while significantly less TZ lesions were assigned to category 2. Differences in GS ≥ 7 tumor frequencies were not significant. Note: Data in parentheses are absolute values and 95% confidence interval. Statistical differences were analyzed with pairwise comparisons using the two-proportions Z-Test. *Results were declared to be significant if *p* < 5%.

Specificity in TZ lesions was significantly lower in v2.1 (Table 3). Specificity for the PZ as well as sensitivity, PPV and NPV for both zones did not differ significantly in both versions.

**Table 3:** Diagnostic sensitivity, specificity, positive predictive value (PPV) and negative predictive value (NPV) for detection of Gleason Score ≥ 7 tumor were calculated for PI-RADS version 2.0 and 2.1.

	PZ			TZ		
	PI-RADS v2.0 (%)	PI-RADS v2.1 (%)	*p* value	PI-RADS v2.0 (%)	PI-RADS v2.1 (%)	*p* value
Sensitivity	95.4	94.3	1.00	95.8	93.8	1.00
Specificity	31.2	24.2	0.10	53.7	42.1	0.03*
PPV	48.8	46.1	0.61	51.1	45.0	0.40
NPV	90.9	86.1	0.51	96.2	93.0	0.50

PI-RADS assessment categories were dichotomized using a predefined cut-off value: a PI-RADS category of ≥ 3 was defined as positive. Specificity in the TZ was significantly lower for PI-RADS version 2.1. All other diagnostic values did not differ significantly. *Results were declared to be significant if *p* < 5%.

### Per-lesion analysis

**Figure 4:**
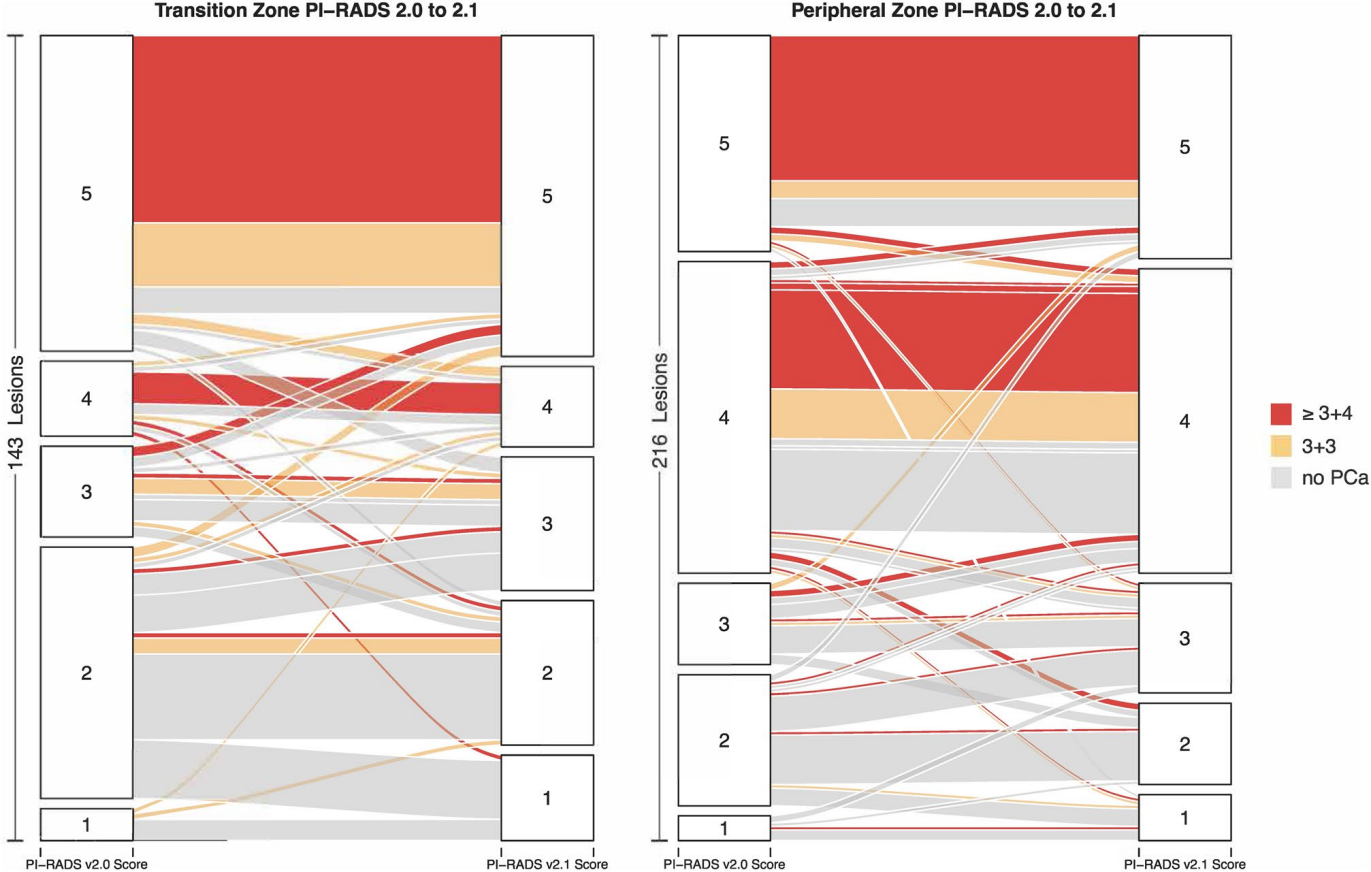
Alluvial plots depicting the change from PI-RADS v2.0 assessments to v2.1 assessment for peripheral (left) and transition zone (right) lesions. Lesion biopsy status is encoded as follows: red: clinically significant (Gleason ≥ 3 + 4), orange non-significant (Gleason 3 + 3) and grey no cancer. Cancer frequencies in category 4 and 5 in PZ and in category 5 in TZ were high in both versions. Variation between the two scoring systems is seen especially in lower scores 1–3. In the PZ, 11 formerly category 2 lesions were upgraded to category 3 in PI-RADS v2.1. Among these only one GS ≥ 7 tumor was confirmed. In the TZ, 11 TZ lesions were downgraded from category 2 into category 1 in v2.1, all of which showed no tumor upon biopsy. 12 formerly category 2 lesions in the TZ were upgraded to category 3 in v2.1, one of them was a GS ≥ 7 cancer. *V* version, *PZ* peripheral zone, *TZ* transition zone, *GS* Gleason Score.

**Figure 5:**
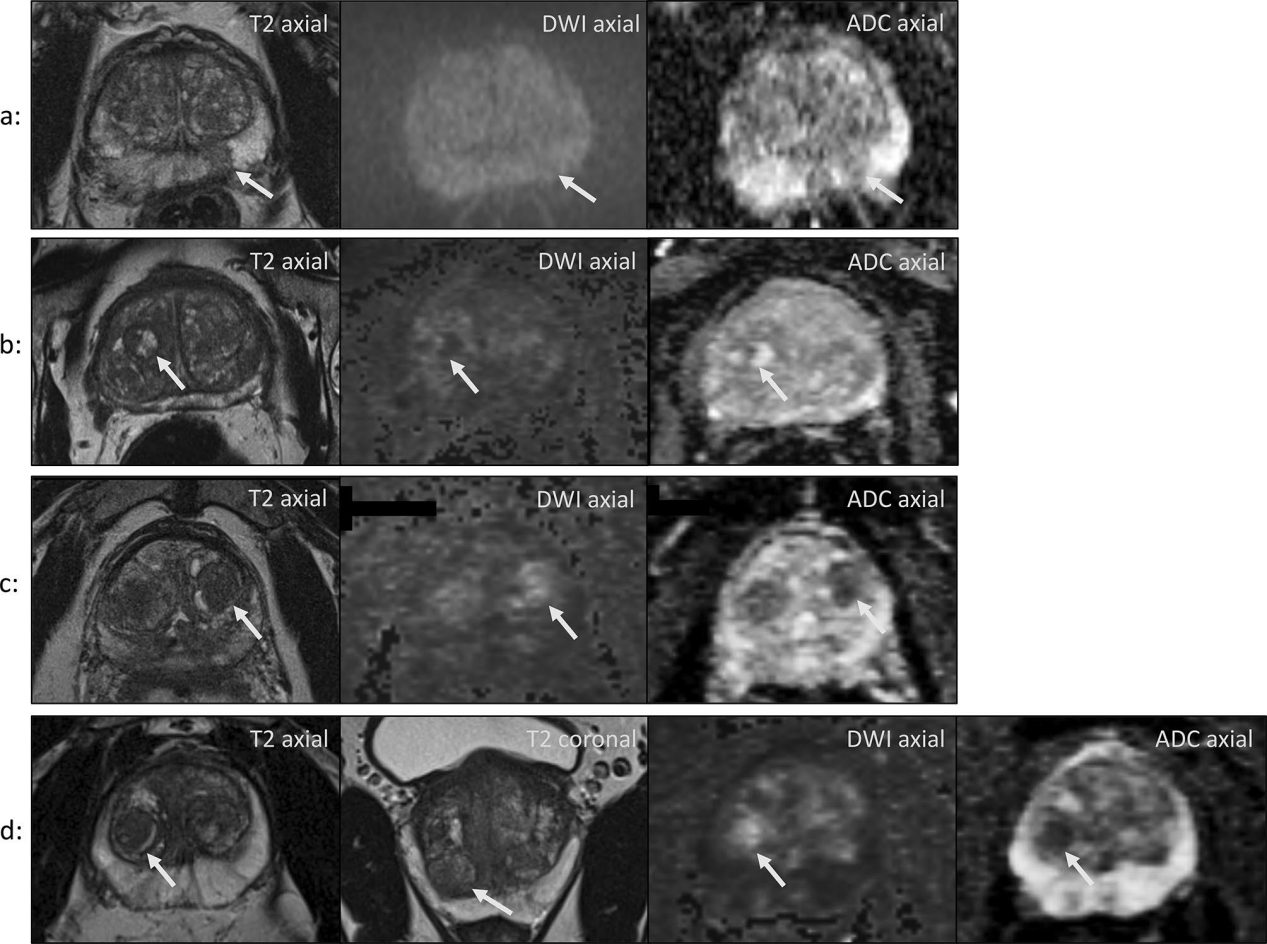
(**a**) Oval-shaped lesion in the left PZ with DWI isointensity and mild indistinct ADC hypointensity. It was scored a 2 according to PI-RADS v2.0 and a 3 according to PI-RADS v2.1. TB of this lesion revealed no cancer. Patient age: 68 years. **(b)** Fully encapsulated and heterogeneous nodule in the right TZ with a diameter of 1.5 cm. It shows heterogeneous signal on high b-value DWI and on ADC images. It was scored into category 2 according to PI-RADS v2.0 and into category 1 according to PI-RADS v2.1 and showed no cancer upon systematic biopsy. Patient age: 56 years. **(c)** Incompletely encapsulated nodule in the left TZ with marked DWI hyperintensity and marked ADC hypointensity. Patient age: 59 years. **(d)** Lesion in the right apical TZ with the same characteristics as in c. Patient age: 52 years. The new DWI upgrading rule was applied to lesions in c and d. In both cases systematic biopsy showed no cancer.

The change of scoring on a per lesion basis between the two PI-RADS versions is visualized using alluvial plots in Fig. 4. The alluvial plot for PZ lesions illustrates that a substantial number of formerly category 2 lesions in the PZ were upgraded to category 3 in PI-RADS v2.1 (n = 11). Among these only one GS ≥ 7 tumor was confirmed (9.1%). Figure 5a illustrates an example. In addition, as depicted in the alluvial plot (Fig. 4), a noticeable amount of TZ lesions scored as category 2 in v2.0 were downgraded into category 1 in v2.1. Specifically, this applied to 11 out of 84 lesions, all of which showed no tumor upon biopsy (see Fig. 5b for an example). Furthermore, 12 formerly category 2 lesions in the TZ were upgraded to category 3 in v2.1 (Fig. 4). The newly introduced DWI upgrading rule in v2.1 has contributed to this change; it was applied to 6 lesions out of 33 TZ lesions with a T2W score of 2, resulting in an upgrade to an overall score of 3 due to a DWI score of 4 or 5 (see Fig. 5c,d for two examples). None of these 6 lesions corresponded to GS ≥ 7 tumors upon systematic biopsy (0%).

### Proposed DWI downgrading decision rule

A proposed decision rule recommending a downgrade of TZ lesions with a T2W score of 3 and a DWI score of ≤ 2 to an overall score of 2 was evaluated: In this study, 6 lesions out of 23 lesions with a T2W score of 3 were applicable to this decision rule and none of these lesions yielded GS ≥ 6 tumor upon biopsy.

### Segment model

The newly introduced segments (left and right posteromedial PZ segment in the base) in v2.1 were marked in 31 of the lesions (9.06%). In all of these lesions neighboring segments were additionally marked.

## Discussion

This study aimed at comparing the diagnostic performance of the recently released PI-RADS v2.1 to its predecessor version 2 using TB and systematic biopsy results as a reference. Our data demonstrate similar AUC and per-score frequencies of GS ≥ 7 tumor in both versions with no statistically significant differences. The distribution of GS ≥ 7 tumor per score in this cohort are comparable to those reported for PI-RADS v2.0 in other studies^11,20,21^.

For the TZ we found very similar AUC for v2.0 and v2.1 with no significant differences. In a recently published study investigating TZ lesions in 58 patients read retrospectively by two radiologists, AUC for v2.1 was slightly higher without statistical significance (0.786 vs. 0.847 for reader 1, and 0.808 vs. 0.858 for reader 2)^22^. A reason for this different outcome may be the different study design in which only TZ lesions that were suspicious based on v2.0 were evaluated. With fundamental changes in the definitions of T2W-based scores 1 and 2 for TZ lesions in v2.1, some stirring up in lesion distributions in categories 1 to 3 could be expected. In v2.0 typical BPH-nodules that show a round shape and complete encapsulation were assigned to category 2, while in v2.1 they are scored into category 1. Likewise, in this study a significant number of TZ lesions scored as category 2 in v2.0 were downgraded into category 1 in v2.1, all of which showed no tumor upon biopsy (0/11). This change constitutes an important improvement in the new version; typical BPH nodules are highly unlikely to be GS ≥ 7 cancer and accordingly are now classified as category 1 lesions for which biopsy is typically not advised. Although this change may not yield a change of outcome for the majority of patients, since neither PI-RADS 1 nor 2 lesions are usually biopsied in practice, it improves the conclusiveness of the radiological report and can prevent unnecessary biopsies in patients with high clinical risk factors and without suspicious MR lesions.

Another significant change in PI-RADS v2.1 is the newly introduced DWI upgrade of T2W score 2 to an overall score of 3 in TZ lesions. In this study, this new upgrade was applied to only 6 lesions out of a total of 143 TZ lesions (4.2%). The impact of the new upgrading rule could thus be minor. Furthermore, none of the upgraded lesions yielded GS ≥ 7 tumor. These upgraded lesions may have contributed to the fact, that specificity in the TZ was lower for v2.1 in this study, with a higher rate of false positives. These results may, however, not be reliable due to the retrospective nature of the study with the possibility that these lesions might have been missed upon systematic biopsy and the fact that lesions were initially identified using PI-RADS v2.0 as well as the small sample size of 6 lesions.

Furthermore, we evaluated a proposed decision rule for TZ scoring: Lesions with a T2W score of 3 and a DWI score of 1 or 2 could be downgraded to an overall score of 2. In this study, 6 lesions were applicable to this decision rule and none of these lesions yielded GS ≥ 6 tumor. Unnecessary biopsies in these patients could have been prevented with this decision rule. With only a small number of cases, this proposed decision rule needs validation in a larger cohort.

In our study, AUC for PZ lesions was slightly lower in v2.1 than in v2.0. To date, there is no literature published concerning diagnostic accuracy of v2.1 in the PZ that our data could be compared to. The modified definition of DWI score 2 might have contributed to the lower AUC of v2.1 found in this study. In v2.0, “indistinct hypointense on ADC” lesions are scored into DWI category 2, whereas in v2.1 lesions of this category should be “linear/wedge shaped hypointense on ADC and/or linear/wedge shaped hyperintense on high b-value DWI”^9^. The added specification regarding the shape of these lesions has led to an upgrading of oval or round shaped lesions in the PZ to a score of 3. These specific lesions showed an unfavourably low frequency of GS > 7 tumor (9.1% or 1/11) in this study.

Additionally, we noticed a small inconsistency with the new DWI score 2 definition that is applicable for all zones. The above mentioned, new criteria describe the appearance of prostatitis in the PZ, despite the fact that prostatitis presents differently in the TZ. Additional clarification regarding DWI score 2 in the TZ could thus be provided in the future.

Regarding the segment model of the prostate, two new segments have been introduced in PI-RADS v2.1: the left and right posteromedial PZ segment in the base. Nine percent of the investigated lesions in this study extended through this newly defined segment on either side but none of these were limited to this specific segment. While the addition of these segments can further specify the localization of a lesion, we did not identify any lesions in this cohort that would have benefited substantially from a biopsy in this specific segment.

There are a number of limitations to this study. The standard of reference was histopathologic results of TB based on the initial read (done prior to the study) and systematic 10-core biopsy. A more reliable standard of reference would be histopathology after surgical prostatectomy, which however would bias the underlying collective towards medium-aggressive cancers. In addition, due to the retrospective design of the study some lesions identified by the readers were not targeted in the biopsies taken ahead of the re-read and could have been missed in the systematic biopsies. Generally, cancer detection rates are higher when TB and systematic biopsy are combined, but the rate of missed GS ≥ 7 tumor in systematic biopsy only is acceptable; Rouvière et al.^23^ reports in a large, prospective, multicentre study, that GS ≥ 7 tumor would have been missed in 7.6% (95% CI 4.6–11.6%) of patients, had TB not been done. Furthermore, readers in this study marked lesions on basis of v2.0 so that in the second review session evaluation was limited to lesions marked in the first review. AUC of v2.1 could thus be biased, e.g. the number of BPH nodules with marked restricted diffusion could be underestimated. Moreover, 45.6% of the included patients received only biparametric MRI without DCE, thus limiting reliability of results in the PZ in category 3 and 4. Furthermore, the readers in this study worked at the same institution, and each lesion was assessed once by a single reader. Interobserver agreement was not investigated in this study. It was previously shown to be moderate for PI-RADS v2.0^5,24^. At the time of publication only two studies have addressed interobserver agreement of v2.1 finding a substantial agreement^22,25^. Lastly, although this analysis is based on a large patient cohort, subgroup analysis is underrepresented.

## Conclusion

The adoption of version 2.1 did not yield significant differences in diagnostic performance regarding per-score frequencies of GS ≥ 7 prostate cancer and ROC-AUC. This is in line with the objective of version 2.1 as stated in the original document^9^. PI-RADS version 2.1 has been mainly created to clarify certain assessment criteria and improve inter-reader agreement while maintaining the framework of version 2. In this study, we find no objections to implementing version 2.1 with regards to its overall diagnostic performance. However, in the TZ, we found significant reduction of category 2 lesions in favor of category 1, corresponding to typical BPH nodules with a favorably low frequency of prostate cancer. Meanwhile, the newly introduced DWI upgrade of T2W score 2 lesions in the TZ was applied only in a few cases, which questions the impact of the new upgrading rule.

## Supplementary information

Supplementary Information.
