# Supplementary material for: Diagnostic performance of PI-RADS version 2.1 compared to version 2.0 for detection of peripheral and transition zone prostate cancer
Source: Sci Rep. 2020 Sep 29;10:15982. doi: 10.1038/s41598-020-72544-z (PMC7525456; doi:10.1038/s41598-020-72544-z)
Supplement: Supplementary file 1 — Supplementary Information. [file 41598_2020_72544_MOESM1_ESM.docx]

**Title: Diagnostic performance of PI-RADS version 2.1 compared to version 2.0 for detection of peripheral and transition zone prostate cancer**

Authors: Madhuri Monique Rudolph, Alexander Daniel Jacques Baur, Hannes Cash, Matthias Haas, Samy Mahjoub, Alexander Hartenstein, Charlie Alexander Hamm, Nick Lasse Beetz, Frank Konietschke, Bernd Hamm, Patrick Asbach, Tobias Penzkofer

# Supplementary tables

**Supplementary Table 1:** Per-reader ROC-AUC for Gleason Score ≥7 tumor detection in PI-RADS v2.0 and v2.1 regarding overall PI-RADS scores per lesion and subscores for T2WI and DWI. Differences in area under the ROC curves (AUC) were not significant. v: version. T2WI: T2 weighted imaging. DWI: diffusion weighted imaging.

|  | | Version 2.0 | | | Version 2.1 | | |
| --- | --- | --- | --- | --- | --- | --- | --- |
|  |  | Overall score | T2W score | DWI score | Overall score | T2W score | DWI score |
| PZ | Reader 1 | 0.76 | 0.79 | 0.78 | 0.77 | 0.79 | 0.76 |
|  | Reader 2 | 0.73 | 0.70 | 0.73 | 0.69 | 0.70 | 0.68 |
|  | Reader 3 | 0.80 | 0.82 | 0.82 | 0.74 | 0.72 | 0.76 |
| TZ | Reader 1 | 0.82 | 0.82 | 0.81 | 0.82 | 0.83 | 0.76 |
|  | Reader 2 | 0.87 | 0.87 | 0.85 | 0.87 | 0.88 | 0.81 |
|  | Reader 3 | 0.69 | 0.69 | 0.71 | 0.69 | 0.69 | 0.69 |

***Supplementary Table 2:*** *ROC-AUC for PI-RADS v2.0 and v2.1 with and without DCE. Differences between mpMRI and bpMRI were not significant. v: version. DCE: dynamic contrast enhanced imaging. mpMRI: multiparametric MRI. bpMRI: biparametric MRI.*

|  | | AUC | p-value |
| --- | --- | --- | --- |
| PI-RADS v2.0 | bpMRI | 0.70 | 0.09 |
|  | mpMRI | 0.81 |  |
| PI-RADS v2.1 | bpMRI | 0.71 | 0.41 |
|  | mpMRI | 0.76 |  |

# Supplementary figures


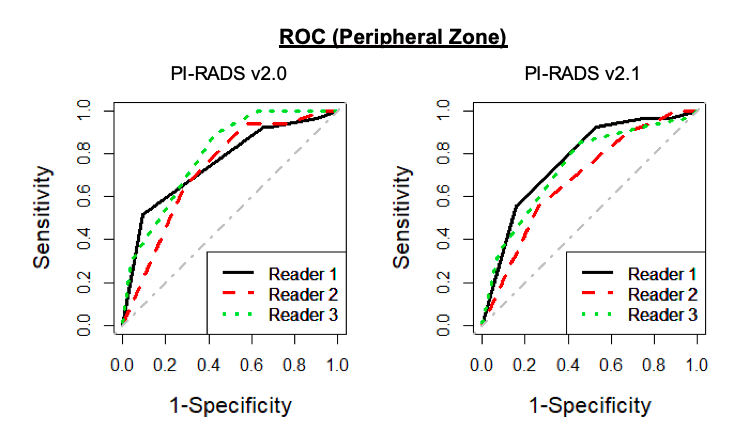


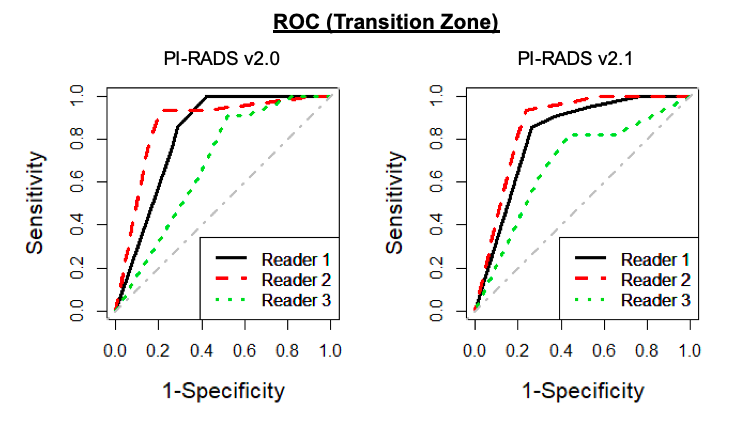


**Supplementary Figure 1:** Per-reader ROC for Gleason Score ≥7 tumor detection in PI-RADS v2.0 and v2.1 regarding overall PI-RADS scores per lesion. Differences in area under the ROC curves (AUC) were not significant. v: version.


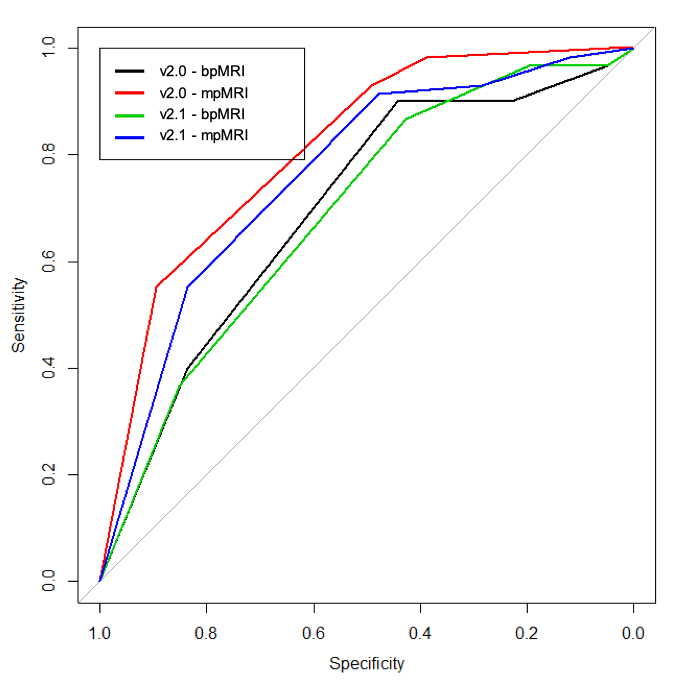


**Supplementary Figure 2:** ROC for PI-RADS version 2.0 and 2.1 with and without DCE. Differences in area under the ROC curves (AUC) were not significant. v: version.
